# Releasing the Immune System Brakes Using siRNAs Enhances Cancer Immunotherapy

**DOI:** 10.3390/cancers11020176

**Published:** 2019-02-03

**Authors:** Mouldy Sioud

**Affiliations:** Department of Immunology, Institute for Cancer Research, Oslo University Hospital-Radiumhospitalet, Montebello, N-0310 Oslo, Norway; Mouldy.Sioud@rr-research.no or Mouldy.Sioud@gmail.com; Tel.: +47-22-78-14-14

**Keywords:** dendritic cells, immunotherapy, gene silencing, adoptive cell therapy, RNA interference, targeted therapies, checkpoint inhibitors

## Abstract

Therapeutic dendritic cell (DC) cancer vaccines rely on the immune system to eradicate tumour cells. Although tumour antigen-specific T cell responses have been observed in most studies, clinical responses are fairly low, arguing for the need to improve the design of DC-based vaccines. The incorporation of small interfering RNAs (siRNAs) against immunosuppressive factors in the manufacturing process of DCs can turn the vaccine into potent immune stimulators. Additionally, siRNA modification of ex vivo-expanded T cells for adoptive immunotherapy enhanced their killing potency. Most of the siRNA-targeted immune inhibitory factors have been successful in that their blockade produced the strongest cytotoxic T cell responses in preclinical and clinical studies. Cancer patients treated with the siRNA-modified DC vaccines showed promising clinical benefits providing a strong rationale for further development of these immunogenic vaccine formulations. This review covers the progress in combining siRNAs with DC vaccines or T cell therapy to boost anti-tumour immunity.

## 1. Introduction

Dendritic cells (DCs) are specialized antigen presenting cells (APCs) derived from bone marrow precursors and are abundant at the skin and internal and mucosal surfaces, where they sense and sample the environment for self- and non-self antigens [1]. Moreover, they bridge innate and adaptive immunity by interacting with a large number of different cell types. Several subpopulations of DCs have been described to date, including myeloid DCs, plasmacytoid DCs Langerhans cells, and monocyte-derived inflammatory DCs. In human, blood myeloid DCs can be divided into CD1c+ and CD141+ DC subsets [1]. While plasmacytoid DCs are well equipped to produce type- I-inteferons in response to infections, CD141+ DC and Langerhans cells are efficient in cross-presentation of cellular antigens and can prime naïve CD8+ T cells to differentiate into effector cytotoxic T lymphocytes (CTLs) [1]. The capacity of DCs to respond to many stimuli is reflected by the expression of a large panel of pathogen recognition receptors, including Toll-like receptors, nucleotide-binding oligomerization-domain receptors and RIG-1-like helicases [1,2]. Additionally, they express various cell surface C-type lectins, chemokine receptors, cytokines and other key regulatory factors such as interleukin- (IL)-12, which drives the generation of CTL responses and enhances the activation of cytotoxic natural killer (NK) cells [2]. As indicated above, DCs can present exogenous antigens, which are usually presented by major histocompatibility complex MHC class II, in conjunction with MHC class I molecules (cross-presentation), resulting in the induction of CTL responses against tumour cells and also against pathogens that do not infect DCs [1,2]. Hence, the development of antitumour immunity relies mainly on presentation of tumour antigens and stimulation by DCs.

**Figure 1:**
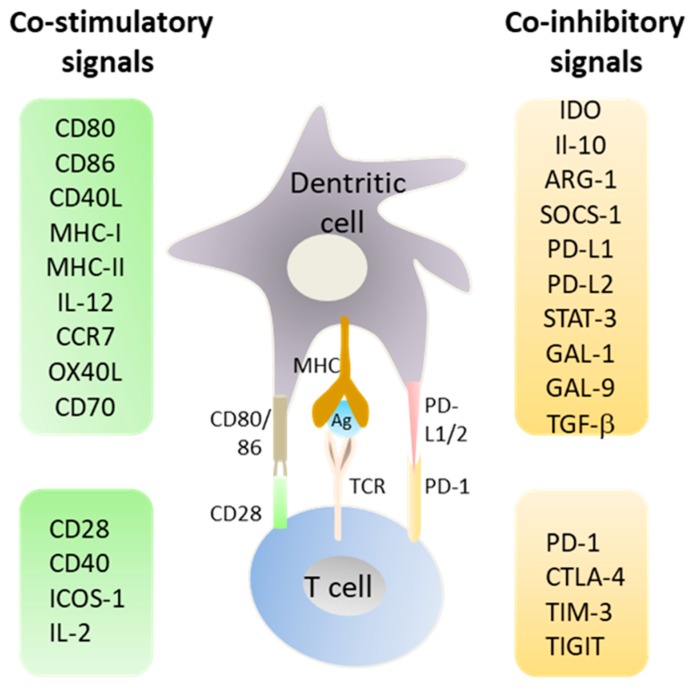
Important role of dendritic cells in deciding immunity versus tolerance. Dendritic cells express several co-stimulatory factors (e.g., CD80, CD86, CD40, IL-12, CCR7) that facilitate T cell activation. To regulate the magnitude of immune responses, dendritic cells DCs also express co-inhibitory molecules such as programmed cell death protein 1 (PD-1) ligands, indoleamine 2,3-dioxygenase ( IDO), and IL-10. Antigen presentation by DC without an additional co-stimulatory signal (e.g., CD80/CD86), or in combination with a co-inhibitory signal (e.g., PD-L1/2) can lead to tolerance induction. Studies in the field of T cell regulation identified the cytotoxic T lymphocyte-associated antigen 4 (CTLA-4) and PD-1 as inhibitory pathways that restrain T cells from full and persistent activation and proliferation under normal physiologic conditions.

Under physiological conditions, the activation of immature DCs by microbial- or certain host-derived molecules, known as danger-associated molecular patterns, induces DC maturation, a process that lead to the expression of co-stimulatory molecules CD80, CD86 and CD40 that are necessary for immune activation; otherwise DCs remain in an immature or semi-mature state that can induce immune tolerance to the captured antigen (Figure 1). Depending on the nature and time course of activation, DCs can express co-inhibitory molecules such as programmed cell death ligand 1 (PD-L1) (B7-H1, CD274) and PD-L2 (B7-DC, CD273) [3,4,5]. The expression of these cell surface molecules, known as checkpoint inhibitors, by DCs is part of the normal function of the immune system in order to protect host tissues from collateral damage during responses to pathogens (Figure 1). In addition to cell surface receptors, DCs also control the strength and duration of immune responses through the expression of cytokines such as interleukin IL-10, transforming growth factor-β (TGF-β), and metabolic enzymes including arginase-1 (ARG-1) and indoleamine 2,3-dioxygenase (IDO) [6,7,8]. Usually, TGF-β is expressed by immature DCs, and its expression decreases upon cell maturation and activation. Human myeloid CD1c+ DCs express higher TGF-β levels compared to CD141+ DC subset [9]. Human plasmacytoid DCs also express significant levels of TGF-β.

**Figure 2:**
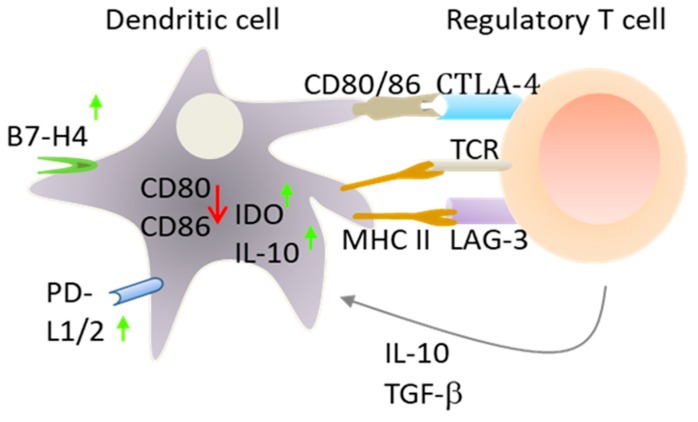
Induction of telerogenic DCs by Treg cells. A number of different factors/signals delivered by Treg cells might function in concert to convert immunogenic DCs into tolerogenic DCs. In addition to cell-cell interactions via membrane receptors, Treg cells can produce IL-10 and TGF-β, which inhibit the function of DCs and therefore the generation of effector T cells (see text). TCR: T cell receptor, LAG-3: lymphocyte activation gene 3, IL-10: interleukin 10.

In addition to intrinsic regulators, DCs also respond to negative signals that are delivered by other immune cells, particularly Treg cells (Figure 2). Among the signals, IL-10 and TGF-β produced by Tregs dampen the antigen-presenting capabilities of DCs by inhibiting the expression of MHC and co-stimulatory molecules [6,10]. In contrast to T cells, Treg cells constitutively express high levels of cytotoxic T lymphocyte-associated antigen 4 (CTLA-4). The binding of CTLA-4 to CD80 and CD86 on DCs downregulates their expression and induces the expression of immunosuppressive factors such as IDO and B7-H4 in DCs [11]. B7-H4 (VTCN1) is a ligand for inhibitory coreceptors on T cells involved in antigenic tolerization. Additionally, the binding of lymphocyte activation gene 3 (LAG3) protein on Treg cells to MHC II molecules on immature DCs activates an inhibitor signaling pathway that suppress DC maturation. This cross-talk between Tregs and DCs is important for the maintenance of peripheral tolerance [10,11]. Notably, Treg cells can develop either during normal development in the thymus, and are then known as thymic/natural Tregs, or can be generated from naïve CD4+ T cells (inducible Tregs) by tolerogenic DCs and are known as inducible Tregs [12]. Mesenteric lymph node CD11b^−^ CD103^+^ PD-L1^++^ DCs highly induce Treg cells [10]. This is probably due to efficient production of the transforming growth factor TGF-*β* and retinoic acid, an active metabolite of vitamin A.

Although the potent capacity of these negative mechanisms to protect the host from autoimmunity and tissue damage has been well established, they might suppress antitumour immunity where sustained T cell activation and proliferation are important [2,5]. Hence, several co-inhibitory signals like those transmitted by cytotoxic T lymphocyte-associated antigen 4 (CTLA-4, CD152) interaction with B7 molecules (CD80/CD86) or those involving the interaction of programmed cell death protein 1 (PD-1, CD279) with its ligands PD-L1 and PD-L2, should be taken into consideration during DC vaccine and adoptive cell therapy (ACT) design. CTLA-4 is a CD28-related protein expressed by activated T cells that interacts with CD80/CD86, but plays an opposing role to that of CD28 causing the suppression of previously activated T cells [4]. Similarly, the interaction of PD-1 expressed by activated T cells with its ligands PD-L1 and PD-L2 on surface DCs leads to inhibition of T cell activation. Both PD-1 ligands are upregulated in response to inflammatory cytokines such as interferon (INF)-γ and IL-10. PD-L1 appears to be overexpressed in various cell types, including tumour cells, whereas PD-L2 is more usually overexpressed in DCs [13]. Given the role played by DCs and T cells in tumour immunity, the current engineering strategies for DC cancer vaccines and ACT should include inhibitors against immune suppressive cytokines, checkpoint ligands, and other suppressive factors such as IDO and ARG-1. The present review highlights the generation of immunostimulatory DCs and functional cytotoxic T lymphocytes using siRNAs to boost anti-tumour immunity. Moreover, it gives a short overview on the therapeutic potential of cancer vaccination that do not relay on ex vivo DCs.

## 2. RNA Interference

**Figure 3:**
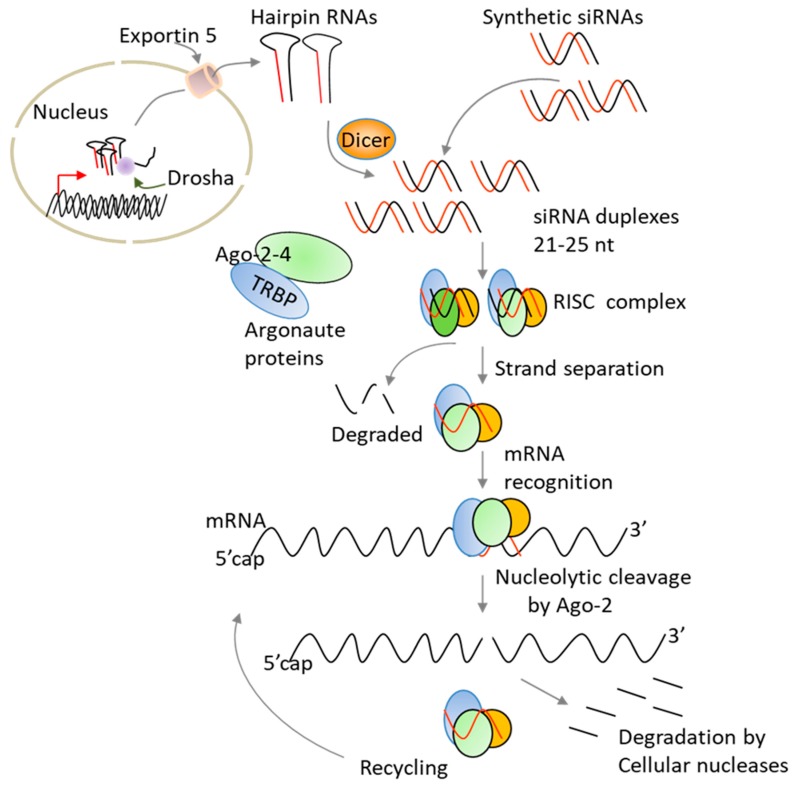
Schematic representation of gene silencing by siRNAs. Synthetic siRNAs are directly loaded into a multi-protein complex termed RNA-induced silencing complex (RISC) where the sense strand with high 5’-end stability is cleaved by the nuclease Argonaute 2 (Ago-2), resulting in strand separation. Subsequently, the RISC containing the antisense strand binds to complementary mRNA sequences. Gene silencing is a result of nucleolytic degradation of the targeted mRNA by Argonaute 2, a RNase H enzyme. Cleaved mRNA molecules are rapidly degraded by cellular nucleases. Following dissociation, the RISC is able to recycle and cleave additional mRNA molecules. Unlike chemically made siRNAs, hairpin RNAs (siRNAs) produced from plasmid vectors in cell nucleus are processed by Dicer in the cytoplasm before entering the RNAi pathway. Normally, hairpin RNAs and microRNAs are processed in the nucleus by the endonuclease Drosha prior to export to the cytoplasm by exportin 5. TRBP: TAR RNA-binding protein.

Since its discovery, RNA interference (RNAi) has emerged as a powerful method for silencing specific genes [14,15]. The technology works by cleaving messenger RNA before it is translated into a protein. As compared to other nucleic acid-based strategies, siRNA benefits from harnessing endogenous RNAi pathway to trigger gene silencing [16]. Two main strategies have been used to harness the RNAi pathway for silencing gene expression: treatment with synthetic siRNA molecules or the expression of short-hairpin RNAs that are processed intracellulary into active siRNAs (Figure 3). Chemically made siRNAs can efficiently silence gene expression without altering the host genetic material. In addition, the delivery of synthetic siRNAs can be altered based on the specific clinical needs, and the treatment can be discontinued, as warranted, without long-term effects. In contrast to antibodies, siRNAs offer a wide ability to selectively target the “undruggable” human genome [14,15].

With respect to therapy, a significant progress has been made in the fields of cancer and viral infections and a variety of carriers including liposomes, lipid nanoparticles, aptamers, and antibodies have been used to deliver siRNA molecules in vitro and in vivo [17,18]. These delivery carriers need to protect naked siRNAs from degradation and avoid fast renal filtration as well as uptake by the mononuclear phagocyte system (macrophages and liver Kupffer cells). In the case of cancer, siRNA-formulations must traffic to the site of tumours and penetrate into and distribute throughout tumour tissues. Unfortunately, several tumours are thought to be more fibrotic and more difficult to penetrate physically. After reaching the target cell/tissue, the siRNA-formulations need to be internalized, escape from the endosomes into the cytoplasm, and finally release the siRNA molecules to enter the RNAi pathway (Figure 3). Although some improvements have been made, the current clinically tested carriers showed shorter circulation times (half-lives < 2 h) [17]. While delivery to hepatocytes is not a major issue for the treatment of liver diseases due to the ingenious use of synthetic N-acetylgalactosamine (GalNAc) ligands [19], delivery to other tissues and organs for therapeutic use of siRNAs requires further improvements. To achieve accurate target gene silencing and successful therapeutic applications, it is also critical to select a target site that has very low sequence homology to human transcriptome in order to reduce off-target effects. Alternatively, one may reduce the silencing of unintended genes by introducing few 2’ chemical modifications in the seed sequence (positions 2–8) of the siRNA guide strand [15,20]. Also, siRNA off-target effects can be reduced by designing siRNAs that work at low concentrations like those that we have developed against IDO [20].

Although the requirement for delivery carriers and endosome escape of siRNA molecules are important for siRNA use in therapy, in case of DC vaccines and ACT, these biological obstacles are insignificant because the cells are modified ex vivo and then re-infused back to the patients [2]. Ex vivo engineered immune cells have also the advantage that their response programs can be systematically corrected and/or improved. Additionally, ex vivo delivery methods of siRNAs would overcome potential off-target effects due to systemic delivery of siRNAs [20]. It should be noted that good manufacturing practice (GMP)-compliant manufacturing protocols for introducing RNAs into DCs or T cells are well established [21,22]. Moreover, the loading of DCs with messenger RNA (mRNA) encoding tumour associated antigens (TAAs) or neoantigens via electroporation is the most used method to introduce mRNA encoding tumour antigens into DCs and it has been tested in several clinical trials [22,23,24,25,26,27]. When delivered via electroporation or nucleofection method, siRNAs effectively silenced gene expression in DCs and T cells without interfering with their immune functions [15,28]. Nucleofection technology, marketed by Lonza (Amaxa®), is an electroporation-based method that is commonly used for the delivery of chemically made siRNAs or plasmid shRNAs into primary immune cells, including peripheral blood T cells and natural killer NK cells.

## 3. Monocyte-Derived DCs

As early mentioned, DCs have unique characteristics that have made them an ideal choice for antitumor vaccines. However, their low number in the blood (around 0.1–1.0% of blood mononuclear cells) limits their direct use in vaccination. Owing to their abundance in peripheral blood, monocytes are an attractive and easily available cell source for DC generation [2,27]. After isolation, the cells can be differentiated into immature DCs by culturing them for 2 to 6 days in the presence of granulocyte-macrophage colony-stimulating factor GM-CSF and IL-4. Subsequently, the immature DCs are exposed to specific maturation cocktails (consisting of various immunostimulatory cytokines and toll-like receptor agonists) for the development of a mature phenotype before injection to patients or cryopreservation [2,20]. Although various types of DC-based vaccines have been generated, the DC-based vaccine most commonly used so far involves the loading of DCs with a source of tumour associated antigens (TAAs) followed by their stimulation with defined maturation cocktails [2,27]. While ex vivo-generated DCs express high levels of co-stimulatory molecules that are required for T cell activation, DC maturation also results in the upregulation of co-inhibitory molecules that may dampen their immunogenic function. Among the inhibitory factors expressed by monocyte-derived DCs are TGF-β, IL-10, PD-L1, PD-L2, suppressor of cytokine signalling (SOCS) 1, and IDO [2,7,28,29]. To escape immune system control, tumour cells also express co-inhibitory molecules and cytokines, including PD-L1, IDO, and HLA-G that has immuno-tolerogenic properties and inhibits CTL and NK cell lytic functions [30,31]. Hence, new strategies for overcoming the immune suppression induced by tumour microenvironment and/or initiated by DC themselves may enhance the immunogenicity and potentiate the efficacy of DC vaccines that are still ineffective in treating cancer patients.

To enhance and sustain DC immunogenic function, one may combine mRNA encoding for tumour antigens with siRNA targeting co-inhibitory molecules using the electroporation method. A potential additional advantage of this delivery method is that multiple siRNAs and mRNA encoding antigens can be loaded onto DCs at the same time. Moreover using transient RNA-based transfection of DC or T cells, sustained severe side effects could be avoided. Once inside the cell, double-stranded siRNAs are extremely stable. Indeed, several studies indicate that dilution due to cell division, and not intracellular siRNA half-life, governs the duration of gene silencing [20]. DCs are relatively stable and do not proliferate. Hence, we expect sustained long-term gene silencing in DC cancer vaccines [28].

## 4. Targeting Indoleamine 2,3-Dioxygenase

Among the immunosuppressive factors that have received attention is IDO, an enzyme involved in the catabolism of tryptophan, an essential amino acid that is required for T cell survival and proliferation, into the stable metabolite kynurenines [32,33,34]. Furthermore, kynurenines can directly generate an immunosuppressive environment by promoting the conversion of CD4+ T cells into Treg cells [32]. Previous work showed that transgenic DCs with high levels of IDO expression and tryptophan metabolites were able to permanently suppress allogeneic T-cell proliferation in vitro [35]. Moreover, IDO was found to be essential for successful allogeneic pregnancy, suggesting that it is important in tolerance induction under normal physiological conditions [32]. Similarly, upregulation of IDO expression in DCs resulted in the induction of a long-lasting allograft tolerance in combination with a locally-restricted immunosuppressive environment [36]. With respect to cancer, the catabolism of tryptophan in tumour cells mediated by IDO has been increasingly identified as a critical microenvironment factor involved in supporting immune escape through suppression of antitumour immunity [37]. Collectively, these results indicate that the depletion of tryptophan by IDO together with an increase in the production of toxic tryptophan metabolites inhibit effector T cell function and induce immune suppressive Treg cells, a major challenge for cancer immunotherapy.

**Figure 4:**
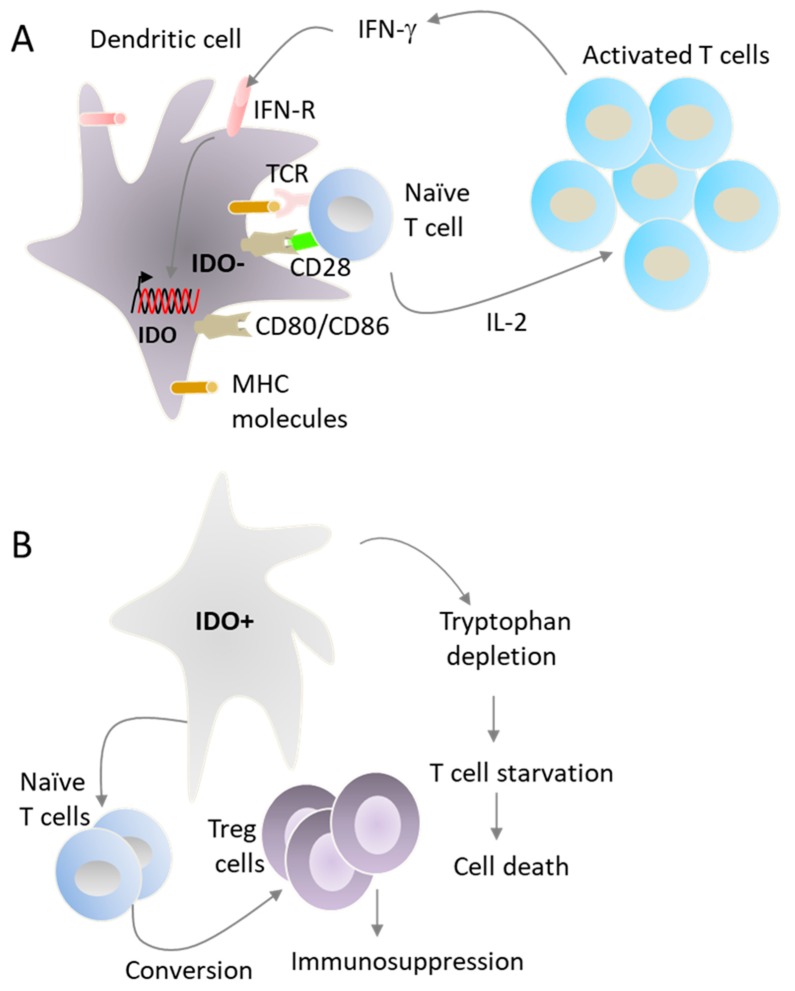
DCs control the duration of antigen-specific immune response. DCs process proteins into antigenic peptides that are presented on major histocompatibility (MHC) molecules to T cells (**A**). The recognition of MHC-peptide complexes by T cells induces antigen-specific signaling via the T cell receptor TCR, known as signal 1. This activation is amplified by the interaction between the co-stimulatory molecules CD80 and CD86 on DC surface with CD28 on T cell surface, known as signal 2, which induces the expression of IL-2. IL-2 facilitates T cell proliferation and clonal expansion. IL-12 produced by DCs promotes the activation of T cells to produce IFN-γ, which favors the generation of CTLs and enhances the activation of cytotoxic NK cells. IFN-γ also induces the expression of IDO by DCs, resulting in their conversion into tolerogenic DCs (**B**). In general, IDO+ DCs polarize naïve T cells into Treg cells, a population of CD4+ T cells that inhibits, rather than promotes, immune responses. Moreover, IDO expressing DCs convert tryptophan into toxic metabolites with immunosuppressive activity on lymphocytes.

In addition to T cell-priming, DCs also control the quality and the duration of T cell activation via the production of cytokines [10]. Once naïve T cells are effectively primed in the lymph nodes, pro-inflammatory cytokines such as interferon (INF)-γ secreted by activated T cells induces IDO expression in DCs (Figure 4A) [32,35], resulting in their conversion into tolerogenic DCs and termination of T cell priming (Figure 4B). Ligation of CD80/86 on DCs with CTLA-4 on T cells also induces IDO expression [38]. In the case of DC cancer vaccines, IDO expression can occur during in vitro maturation of monocyte-derived DCs as well as in vivo after T cell activation [28,39]. Under our ex vivo culture conditions, all DC maturation cocktails induced IDO [40,41], thus highlighting the urgent need for the identification of new therapeutic agents that can enhance the immunogenic function of DC cancer vaccines.

A strategy for enhancing the potency of DC cancer vaccines would be the blockade of IDO expression in DCs prior to re-infusion into patients. We have previously demonstrated that DCs modified with siRNAs against IDO strongly activated T cells as compared to their unmodified counterparts [28]. Moreover, IDO-silenced DCs expressed high levels of co-stimulatory molecules similar to control DCs, indicating maintenance of their mature and stimulatory phenotype. In preclinical studies, Zheng et al., showed that an efficient anti-tumour response can be induced with an IDO-silenced DC vaccine in a syngeneic mouse model for breast cancer [42]. The vaccination resulted in the induction of tumour antigen-specific CTL responses and decrease in the number of circulating Treg cells.

Having demonstrated that IDO-silenced DCs are functional in vitro, next we have investigated the feasibility, safety, and immunogenicity of the vaccine in four patients with metastatic ovarian cancer using our GMP-compatible DC production pipeline [21,43]. Fast DC preparations from patients were transfected ex vivo with IDO siRNA along with mRNA encoding the human reverse transcriptase subunit of telomerase (hTERT) or survivin using the standard electroporation method [21]. After, the cells were grown for 24 h with cytokines facilitating maturation (TNF-α, IL-6, and prostaglandin E2) and then cryo-preserved into separate vaccine doses. It should be noted that hTERT is an attractive, almost universal antigen target in cancer being overexpressed in the majority of human tumours and absent in most normal adult tissues [44]. Moreover, hTERT is expressed in cancer cells with stem cell-like properties and targeting this antigen could therefore be an important tool to eliminate these cells, which are not easily killed by conventional therapy [45]. The engineered ex vivo IDO-silenced DCs activated T cells more effectively than control DCs in allogeneic mixed lymphocyte reactions. Importantly, the clinical data revealed that the vaccine is safe, well tolerated, and has therapeutic potential even in advanced stage ovarian cancer when compared to matured “standard” DC vaccine [43]. Similarly, two metastatic prostate cancer patients who received the IDO-silenced DC vaccine in 2014 showed clinical benefits that correlated with a drop in serum prostate specific antigen (PSA) levels. One of the vaccinated patients is still alive. Collectively, these data provide a strong rationale for further development of IDO-silenced DC vaccine alone or in combination with other immunotherapies.

Since anti-CTLA-4 antibody (ipilimumab) was approved as therapy for the treatment of melanoma patients [46], we addressed the question whether the combination of ipilimumab with our improved DC vaccine could enhance the anti-tumour effects in a patient who initially did not respond to the antibody therapy. The vaccination resulted in a significant anti-tumour effect that was associated with a significant induction of T cell responses against the antigens used for vaccination, namely hTERT and survivin [47]. Moreover, antigen-specific responses to melanoma tumour antigens such as MART-1 and NY-ESO-1 were induced, suggesting that the current combination therapy induced epitope spreading. Overall, the vaccination induced antigen-specific immune responses and clinical benefits. Although the requirement for large and well-designed randomised trials to assess the efficacy of IDO-silenced DC vaccine is clear, case studies can make important contributions as instructive scientific observations.

## 5. Targeting CTLA-4, PD-1, and PD-1 Ligands

Under normal conditions, the stimulation of T cells via the T cell receptor (TCR) leads to intracellular signalling that promotes T cell activation and proliferation [3]. Following activation, T cells express on their surface CTLA-4, a membrane receptor that antagonizes T cell function through inhibition of CD28 signalling by competing for their shared ligands B7 molecules [3,4]. CTLA-4 has a higher affinity for B7 molecules than CD28 and serves as a natural inhibitor to terminate T cell priming by DCs in the lymph nodes. Effector T cells also upregulate the expression of PD-1, a negative regulator of T cell function [5]. Unlike CTLA-4, PD-1 signalling pathway is a checkpoint involved in controlling peripheral tissue damage after inflammatory immune responses. Excessive induction of PD-1 on T cells in the setting of chronic antigen exposure inhibits T cell activation, as a result of T cell exhaustion. Hence, during immunotherapy the interactions between PD-1 and its ligands, PD-L1 and PD-L2, expressed by APCs such as DCs or tumour cells is expected to block the immune responses against tumours. Similar to CTLA-4, the PD-1 signalling pathway is also involved in extrinsic suppression of T cell activation by Treg cells [48].

Agents such as monoclonal antibodies (mAbs) and siRNAs targeting these inhibitory receptors could substantially boost the efficacy of DC vaccines or restore the killing potency of tumour infiltrating lymphocytes (TIL). Indeed, antibodies that bind to either CTLA-4 or PD-1, and thereby alleviate the immune inhibition indicated above, have generated promising clinical data in patients with melanoma or lung cancer [49,50,51,52,53]. However, only a fraction of patients responded durably to the therapy. Furthermore, severe side effects were detected in a high proportion of patients, especially in those treated with anti-CTLA-4 therapy as compared to anti-PD1 therapy [53]. These effects are more likely due to the polyclonal activation of autoreactive T cells due to systemic administration of the antibodies. Hence, additional targeted strategies designed to inhibit the expression of these co-inhibitory molecules are warranted. In this respect, Hobo et al. used RNAi to downregulate the expression of PD-1 ligands in DCs [54]. PD-L1 and PD-L2-silenced DCs enhanced T cell proliferation in vitro. Similarly, silencing PD-L1 in DCs enhanced the anti-tumour effects of bladder cancer antigen-loaded DC vaccines [55]. SiRNA silencing of PD-1 ligands on DC vaccines also enhanced the expansion of minor histocompatibility antigen (MiHA)-specific CD8+ T cells in mice [56]. In addition to electroporation, recently the authors used a new cationic lipid formulation (named SAINT-18) that is compatible with GMP manufacturing to deliver PD-L1 and PD-L2 siRNAs to DCs [57,58]. Interestingly, PD-L-silenced DCs loaded with mRNA encoding for MiHA showed enhanced MiHA-specific T-cell activating potential than control DCs. Overall, the data indicate that silencing of PD-1 ligands in DCs can enhance their immunogenic function, leading to the induction of stronger antigen-specific CTL responses in in vitro models and antitumor immunity in various mouse cancer models [59]. Given the encouraging in vitro data, a phase I/II study in currently underway to investigate the clinical effect of a MiHA-loaded PD-L silenced DC vaccine (NCT02528682).

## 6. Targeting SOCS-1 and STAT-3 Transcription Factors

SOCS-1, a member of the suppressors of cytokine signalling proteins SOCS, has also emerged as a critical inhibitory molecule for controlling the responses to cytokines and antigen presentation by DCs. SOCS-1 is induced by cellular activation and serves as a negative feedback mechanism for a range of cytokines. A number of studies in mice have documented the important role of SOCS-1 in modulating the extent of immune stimulation by DCs suggesting that its inhibition may enhance vaccine-induced immune responses. Indeed, silencing SOCS-1 in DCs enhanced antigen presentation, T cell priming, and anti-tumour immunity [60,61]. More recently, Wand and colleagues tested the therapeutic efficacy of a SOCS-1-silenced DC vaccine loaded with two tumour-associated antigens, survivin and MUC-1 in patients with relapsed acute leukemia (AL) after allogenic hematopoietic stem cell transplantation (allo-HSCT) [62]. Relapse after allo-HSCT is the leading cause of treatment failure for AL patients [63]. The designed DC vaccine was tolerated and induced antigen-specific CTL responses capable of selectively killing leukemic cells. Interestingly, clinical data showed that 10 out of 12 early relapsed patients who received the vaccine exhibited complete remission. Moreover, patients treated with the vaccine had no grade 3 or 4 graft-versus-host disease, again indicating that the treatment with the vaccine is safe. Hence, the inhibition of SOCS-1 expression in DCs could be exploited as a novel adjuvant strategy to boost the potency of DC cancer vaccines (NCT01956630). Although this clinical success is very encouraging, defining how the vaccine impacts the durability and memory properties of CD8+ T cells will be critical for understanding the potential of this vaccine to provide a durable mechanism of immunity in setting of cancer relapse.

In addition to SOCS-1, the induction of signal transducer and activator of transcription 3 (STAT-3) in DCs by tumour-derived factors renders DCs tolerogenic and suppresses their antitumour potency. PD-L1 expression in DC is also regulated by STAT-3 signalling pathway [64]. Hence, silencing STAT3 in DCs should be beneficial for cancer immunotherapy. A number of therapeutic strategies have explored selective inhibition of STAT-3 signalling using various agents such as small molecule inhibitors, dominant-negative STAT-3 mutants and siRNAs. Systemic administration of STAT-3 inhibitors not only inhibited tumour growth, but also resulted in the downregulation of immunosuppressive cytokine expression and induction of functional DCs and CTL responses. Similarly, STAT-3 gene silencing restored DC maturation and enhanced CTL responses to tumours [65]. The siRNA strategy was also applied to CD204, an immunosuppressive scavenger receptor in DCs [66]. CD204-silenced DCs were injected into the tumours followed by local radiotherapy, resulting in enhanced anti-tumour effects. Gene silencing of galectin-1 and galectin-3 in DCs also enhanced their capacity to stimulate T cell activation and IFN-γ production [67]. In addition to the regulation of inflammatory responses, galectin-1 and galectin-3 may function as a negative regulator of T cell activation by increasing the TCR activation threshold as both proteins have been shown to interact with CD45, CD7, and CD3 molecules within the immunological synapses [68]. Normal T cells undergo a process of TCR desensitization before entering the secondary lymphoid tissues. This TCR tuning modulates the intensity of TCR signalling and is thought to be especially important for cells with relatively high self-reactivity [69]. In principle, lowering the activation threshold of TCR increases the expansion of T cells, which is beneficial in immunotherapy.

## 7. Targeting IL-10

Although IL-10 is involved in various aspects of the anti-inflammatory process, its main function is related to the downregulation of T cell function [70]. Moreover, IL-10 increases PD-L1 expression in macrophages and dendritic cells. Various stimuli, in particular Toll-like receptor- (TLRs) agonists have been explored as adjuvants in different vaccination strategies or in ex vivo DC maturation cocktails. Although TLR agonists efficiently induce DC maturation and IL-12 expression, they also induce IL-10 expression [71]. Whether produced by DC themselves or present in the tumour microenvironment, IL-10 conditions human DCs to acquire a tolerogenic phenotype favouring the induction of T cell anergy and suppressive function. Moreover, early in vitro studies have identified IL-10 production by DCs to be an important mechanism in driving T cell anergy and suppression [72]. Hence, the development of agents that stimulate DC maturation and simultaneously inhibit the expression of IL-10 may facilitate the design of better DC vaccines.

**Figure 5:**
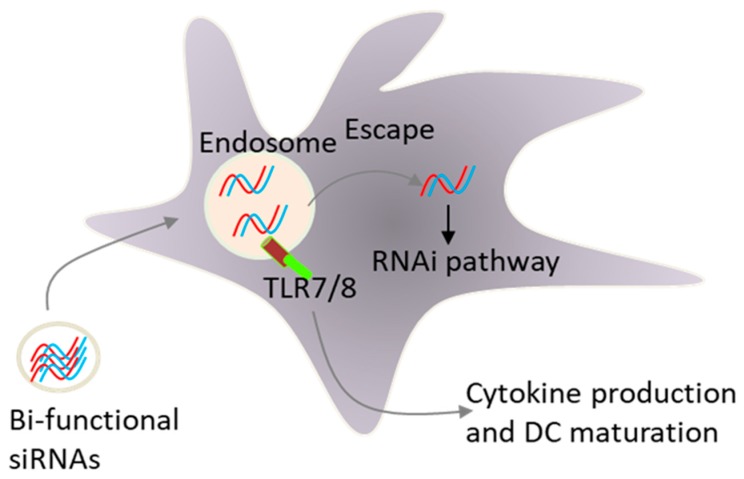
Combined gene silencing and Toll-like receptor TLR activation by a single siRNA sequence. Most siRNA molecules delivered by cationics lipids, nanoparticles or cell-type-specific delivery agents, begin their intracellular trafficking in the endosomal vesicles where they can activate toll-like receptors, resulting in cytokine production and eventually DC maturation. After endosomal escape, siRNA molecules enter the RNAi pathway to exert their silencing function as illustrated in Figure 2.

Early, we and others have demonstrated that the formulation of siRNAs in lipid-based delivery vehicles, which deliver siRNAs to endosomal compartments in which TLR7 and TLR8 reside, activate innate immunity, leading to type I interferon and pro-inflammatory cytokine production [15,73]. These unwanted effects present a key challenge for siRNA therapeutics. However, there are clinical situations, such as viral infection and cancer, in which this adjuvant effect of siRNAs could be beneficial. In fact, TLR activation was more likely responsible for the promising early clinical trial data for ocular anti-angiogenic siRNAs and topically applied siRNAs respiratory syncytial virus [74]. Site specific chemical modifications that reduce sequence-specific off-target effects also inhibited the activation of TLRs by siRNAs [75,76,77,78,79]. In contract to lipid delivery, we demonstrated that siRNAs delivered via electroporation to the cytosol did not activate innate immunity, again supporting the involvement of endosomal TLRs in siRNA sensing by immune cells [73]. Because certain siRNA sequences activated TLR-7 and TLR-8 [73,78], we hypothesized that a combinatorial strategy that blocks the expression of immunosuppressive factors and simultaneously activate innate immunity via TLRs could increase the efficacy of DC vaccines (Figure 5). In this respect, we have shown that targeting IL-10 expression by a bi-functional siRNA can block IL-10 expression and activates TLR-7/8 signalling in human monocytes and monocyte-derived immature DCs [79]. The inhibition of IL-10 and deliberate activation of immature DCs with a single siRNA sequence opens the possibility of generating mature DCs without the addition of external cytokines. This strategy of DC maturation may be extended into a therapeutic vaccination setting. In this respect, we have developed a new vaccination protocol where ex vivo immature DCs were activated with lipid liposomes formulated with tumour antigens and an IL-10 siRNA capable of co-coordinately activating DCs via TLR7/8 and silencing IL-10 expression [80]. Using a rat model of acute myeloid leukemia, we found that the vaccination with these DCs pulsed with liposomes was more effective in stimulating CTL responses against tumour cells when compared to control vaccine. Thus, the in vivo application of a DC vaccine loaded with bi-functional IL-10 siRNA, not only promoted DC maturation and enhanced CTL responses, but also suppressed leukemia cell growth in vivo [80]. Moreover, i.v. vaccination with the liposome-containing tumour antigens and IL-10 siRNA induced type- I- interferon production and cytotoxic T cell responses against tumour cells, further supporting the use of lipid based nano-carrier agents for the activation of DCs in situ as well.

Previous studies have shown that the adjuvant effect of CpG oligonucleotides is TLR-9 dependent [71,81]. In addition to IL-12, CpG DNA motifs also induced the expression of IL-10 and TGF-β in vitro and in vivo. While IL-12 is critical for the orchestration of cellular immunity, both IL-10 and TGF-β promote immune tolerance. Hence, blocking IL-10 at the time of treatment with CpG DNA may enhance anti-tumour effects. In this respect, simultaneous immunotherapy with CpG DNA and IL-10 siRNA enhanced immune protection of an idiotype DNA vaccine in a prophylactic murine model of B cell lymphoma as compared to control treatments [82]. Similarly, IL-10 gene silencing in DCs induced stronger CTL responses against the human melanoma antigen MART-1 as compared to unmodified DCs [83]. More recently, Ahn et al. showed that co-targeting IL-10 receptor and TGF-β in DCs by siRNAs enhanced the immunogenic function of DCs, resulting in a significant anti-tumour effect against highly immune resistant tumour cells that secrete more IL-10 and TGF-β than the parental tumour cells [84]. Other studies also reported that co-administration of IL-10-specific siRNA-loaded nano-carriers and a DC vaccine enhanced antitumor immune responses and inhibited tumour growth and metastasis [85]. Collectively, these findings indicate that targeting IL-10 gene expression with siRNA in DCs before or at the time of immunization can increase vaccine induced CTL responses and block tumour growth in animal models. Hence, combining IL-10 siRNA with DC vaccines should be a strategy of choice to enhance vaccine efficacy. This conclusion is further supported by the fact that clinical and strong immunological responses in prostate cancer patients correlated with low expression of IL-10 in DC preparations [86].

## 8. Targeting INF-γ-Induced Proteasome Proteases

Notably, the development of an effective cancer vaccine requires effective processing and presentation of TAAs to T cells. The standard proteasome is considered to play a central role in peptide generation for MHC class I molecules that present peptides primarily derived from proteins located in the cytosolic compartment of the cell [87]. The catalytic activity in charge for the cleavage of the peptides is exerted by three proteases of the β-ring subunits, namely β1, β2 and β5 [87]. In the presence of IFN-γ, these three catalytic subunits are replaced by their homologous subunits β1i (LMP2), β2i (MECL1) and β5i (LMP7), resulting on the formation of the so-called immunoproteasome that is more efficient at processing antigenic epitopes [87]. Immature DCs usually express both proteasome types, whereas mature DCs express only the immunoproteasome and therefore only present peptides generated by the immunoproteasome. All, non-self proteins including microbial proteins are processed by the immunoproteasome. However, several self-proteins such as melanoma antigens used in DC vaccination are not processed by the immunoproteasome [88]. The induction of the immunoproteasome in mature DCs may be one mechanism to control the development of autoimmunity by inhibiting the processing of self-proteins. Given that most TAA are self-proteins, this safeguard mechanism may limit the effectiveness of DC cancer vaccines. Hence, the knockdown of LMP2, LMP7, and MECL-1 gene expression could be used to enhance cancer vaccine potency.

By targeting the expression of these IFN-γ inducible proteases by siRNAs, Dannull et al., showed that TAAs can be processed into peptides that are more appropriate for the induction of effective anti-melanoma immunity [89]. Although the levels of gene silencing are not remarkable, the proteasome-modified DCs stimulated superior anti-melanoma immunity in vitro. Based on these results, the authors have initiated a phase I clinical trial in which patients with metastatic melanoma were vaccinated with mature autologous DCs transfected with RNAs encoding the malanoma antigens MART-1, MAGE3, gp100, and thyrosinase in combination with siRNAs against the three inducible immunoproteasome subunits indicated above [90]. Vaccination stimulated antigen-specific T cell responses in all patients, which peaked after 3–4 vaccinations and sustained high only in patients who received siRNA-modified DCs. Two patients with active metastatic disease at the time of vaccination with siRNA-modified DCs showed clinical benefits. One patient showed a complete clinical response and remained free of active disease 20 months after the end of vaccination (NCT00672542). Although more clinical data are needed, the data support the use of the proteasome-modified DCs in cancer immunotherapy.

## 9. Vaccinations that do not Relay on Ex-Vivo DCs

### 9.1. Peptide- and Recombinant Protein-Based Vaccines

An alternative strategy to ex vivo generated DCs is vaccination with recombinant tumour antigens or peptides capable of recruiting and activating native DCs in situ. However, such vaccines are often poorly immunogenic and require supplementary components to help stimulate effector T cell functions. Several components, termed adjuvants, provide the “help” needed to enhance the immunogenicity of vaccine antigens. It should be noted that immune adjuvant-based therapies have been used to amplify spontaneous immune responses against TAAs which are rarely sufficient to cause the regression of established tumours. This has become the standard of care for superficial bladder cancer patients with radical surgery followed by intravesicular injection of bacilli Calmette-Guérin which provokes a local inflammatory reaction in the bladder wall [91]. Many adjuvants including imiquimod, a Toll-like receptor 7 agonist, and double-stranded RNA poly I:C, a TLR3 agonist, are used in cancer vaccines [92]. Based on promising clinical responses and low toxicity, polyICLC, a clinical grade modified formulation of polyI:C (stabilized with poly-L-lysine and carboxymethylcellulose) is believed to be a good adjuvant for the induction of CTL responses [92]. Similarly, GM-CSF has been used in clinical management of multiple cancers as a monotherapy as well as an adjuvant with cancer vaccines. GM-CSF increases the recruitment and maturation of DCs, as well as antigen presentation [93]. The current standard cancer vaccination has used recombinant TAAs or peptide in combination with a suitable immunological adjuvant. Such vaccines are usually injected subcutaneously or intradermally and require multiple doses to induce adequate T cell responses. Despite promising preclinical results, the vast majority of the tested vaccines showed limited clinical effects in cancer patients [94,95].

The use of antigenic peptides that originate from somatic mutations known as neoantigens may enhance vaccine potency. Moreover, the simultaneous blockage of immune checkpoints with antibodies or siRNAs may enhance peptide-based vaccinations especially in patients with non-immunogenic tumours. In this respect, Ott et al. reported the results of a phase I trial of a personalized cancer vaccine that targets up to 20 patient neoantigens [96]. In this trial, 6 patients with melanoma (stage III/IV) were vaccinated with 97 T cell neoepitopes delivered by subcutaneous injection as long-peptides in combination with polyICLC as adjuvant (NCT01970358). The vaccine induced tumour-antigen-specific immune responses. Four out of six patients treated showed no recurrence at 25 months, and progressing patients responded to further therapy with anti-PD-1. In addition to somatic mutations, tumour-specific antigens can also be generated as a consequence of viral transformation, as in the case of human papillomavirus type 16- driven oral and cervical tumours [97]. In contrast to TAAs, high affinity T cells against neoantigens and viral proteins are not deleted during thymic selection and therefore vaccination with such antigens is expected to generate high-affiniy immune T cell responses against tumour cells [98]. The FDA has approved Gardasil9 and Cervarix (adjuvanted non-infectious recombinant vaccines) for use in healthy women as prophylactic vaccines against multiple variant of HPV which are associated with the development of cervical carcinomas and anal cancers. These new vaccines will protect against approximately 90% of cervical cancers. Thus, certain standard cancer vaccines can be effective.

### 9.2. Targeting DCs in Situ

Although ex vivo-generated monocyte-derived DCs share many phenotopic and functional characteristics with natural DCs, whether they are the optimal sources of therapeutic DCs remains unclear. As indicated in Section 3, the compounds used for monocyte differentiation into DCs induce the expression of immunosuppressive factors such as IDO. Moreover, vaccines loaded with antigen ex vivo require the vaccine to be tailor-made for each patient. Targeting tumour antigens to DCs in situ would overcome these limitations and moreover the vaccines can be produced in bulk quantities (off-the-shelf-use). In general, tumour antigens are either chemically coupled or genetically fused to antibodies that recognize receptors expressed by DCs [99]. Indeed, early studies from Ralph Steinman’s group demonstrated the principle of targeting antigens to DCs in situ through the coupling of antigens to antibodies that target DC surface receptors involved in uptake such as members of the C-type lectin family including DEC205 and the mannose receptor [100]. A critical finding from these early studies was the induction of tolerance when antigens were not coupled with adjuvants that activate DCs. Similarly, targeting via other DC surface receptors such as DC-SIGN, CLEC9A, DCIR, Dectin-1 and Langerin with combination with adjuvants stimulated humoral and cellular immune responses in preclinical studies [101,102,103]. The first-in human study was reported in 2014 by Dhodakar et al. where patients were vaccinated with the cancer-testis antigen NY-ESO-1 fused to antibody targeting DEC-205 in patients with malignancies known to express NY-ESO-1 [104]. The vaccine was combined with a topical or subcutaneously administrated adjuvant consisting of different TLR agonists. Overall, the vaccine was safe, induced NY-ESO-1-specific immune response, and thirteen out of forty-eight patients had stabilization of disease with median duration of 6.7 months. However, only two patients showed tumour regression. Given the variability of responses among patients in each group, further studies would be required.

Due to their specificity, targeted delivery and low cytotoxicity, biodegradable nano-carriers could lead to more efficient antigen delivery to DCs [105]. DCs pulsed with certain nano-carriers loaded with TAAs were reported to possess higher ability to cross-present antigens and induce strong cytotoxic responses [105]. With respect to in vivo applications, these formulations can function both as a delivery agent to enhance antigen navigation to DCs and as an immunostimulatory adjuvant to activate DCs. The advantage of co-delivery of antigens and adjuvants is that adjuvants only activate those DCs that are targeted by the nano-carriers, thereby preventing systemic activation and toxicity of adjuvants. The nano-carriers can be decorated on their surface with antibodies, carbohydrate ligands, or peptides that bind specifically to DC receptors. Liu et al., showed that nanoparticles can deliver an mRNA vaccine encoding tumour antigen MUC1 to DCs in lymph nodes [106]. Similaly, Shi et al., developed a chitosan nanoparticle loaded with whole tumour cell lysates and decorated with surface mannose moieties for specific DC targeting [107]. The engineered vaccine showed therapeutic effects in mice with melanoma. Although antibody-targeted nano-carriers loaded with TAAs not yet tested in the clinic, a phase I/II study in stage II-IV melanoma patients vaccinated with a melanoma-specific Melan-A/Mart-1 peptide fused to virus-like nanoparticles loaded with A-type CpG, a ligand for toll-like receptor 9, induced T cell responses in 14 out 22 patients [108].

In contrast to antibodies, short peptides would represent an important targeting tool because of their excellent tissue penetration and easy synthesis and conjugation to nanocarriers. We have undertaken a study to test the hypothesis that DC-targeting short peptides selected from phage peptide libraries would increase antigen immunogenicity and enhance T cell priming [109]. One of the selected DC-binding peptides (NW-peptide) bound to macrophages and DCs. The NW peptide was conjugated to chitosan nanoparticles loaded with ovalbumin (OVA) as a model vaccine. We have used chitosan (CH) as polymer matrix because this reagent is particularly suitable for clinical and biological applications owing to its low toxicity, biocompatibility, biodegradability, and low immunogenicity. Flow cytometry and immunocytochemistry studies demonstrated a strong targeting ability of NW-nanoparticles to macrophages and DCs. Preclinical studies are underway.

Most engineered nano-carriers and liposomal delivery systems showed a predominant uptake in the liver tissue where macrophages and DCs reside [105]. Based on these early observations, Kranz et al., developed antigen-encoding RNA formulations (named RNA-lipoplexes) capable of systemic targeting of liver DCs and macrophages without the need for conjugation to targeting ligands [110]. The authors identified mouse CD11c+ DCs in the marginal zone, and plasmacytoid DCs and macrophages in the spleen as the main targeted cells. The engineered RNA-vaccines induced anti-tumour immunity in several preclinical cancer models. Interestingly, a specific antigen-specific T cell response were induced in the first three melanoma patients who received the RNA-lipoplexe vaccine encoding four tumour antigens: NY-ESO-1, MAGE-A3, tyrosinase and TPTE (NCT02410733). The development of this simple vaccination approach in combination with siRNAs targeting checkpoint inhibitors should further enhance clinical responses.

## 10. Enhancing the Functionality of T cells

The generation of T cells with superior cytotoxic activity is essential for developing effective immunotherapeutic strategies against infectious agents and cancers. Similar to DCs, the activation of T cells is negatively regulated by various intrinsic co-inhibitory factors such as CTLA-4, PD-1, and TCR-adaptor proteins (Figure 1). Hence, adoptive cell therapy (ACT) must address these various inhibitory barriers in order to enhance therapy response. ACT is a potent method of harnessing autologous immune cells, allowing for ex vivo manipulation of T cells or natural killer cells prior to their re-infusion into the patients [111,112]. However, ex vivo cultured T cells appear to upregulate the expression of certain inhibitory factors such as CTLA-4 and PD-1, which dampen their in vivo function. Combining ACT with antibodies targeting checkpoint inhibitors is an attractive possibility already pursued in clinical trials [113]. However, such combination may results in systemic serious adverse events caused by the antibodies acting on patient autoreactive T cells. Moreover, cytosolic inhibitory factors are difficult to target with antibodies. An alternative option would be the ex vivo inhibition of these inhibitory factors by siRNAs. As for DCs, a versatile method that could potentially be used for siRNA delivery to T cells is the ex vivo route, whereby blood T cells or TIL could be isolated from a patient, transfected with siRNAs, and then infused into the same patient [111,112]. In our experience the use of standard wave electroporation method can deliver siRNAs to human blood T cells with close to 95% transfection efficiency and with a little effect on cell viability and function. Nucleofection of siRNA into T cells also resulted in significant gene silencing [114,115]. Furthermore, the introduction of chemical modifications into siRNAs extended gene silencing in T cells [114]. Indeed, the authors showed that transfected T cells remain functional and maintain siRNA-induced CD4 knockdown for up to 2 weeks after transfer into recipient mice. With respect to immunotherapy, T cells transfected with siRNAs against PD-1 showed an increased cytotoxic effect against PD-L1-expressing melanoma cells [116]. Similarly, gene silencing of CTLA-4 in T lymphocytes from hepatitis B positive patients resulted in a stronger CTL response against virus-infected cells [117]. These in vitro studies are encouraging and should set the stage for in vivo investigations.

Previous studies have shown that the attachment of a 3′ cholesterol group to the sense strand of siRNA facilitated its uptake by human cells [118]. Chemically modified self-delivered siRNAs provide an alternative RNAi strategy because they enable siRNAs to enter cells without the need of a transfection reagent. This type of siRNAs has been successfully used in RNAi experiments in a variety of primary cells, including macrophages, T cells, and in vivo models, thus holds great potential for clinical applications [119]. More recently, Ligtenberg et al., used this strategy to deliver PD-1 specific siRNA to tumour infiltrating lymphocytes (TIL) [120]. Interestingly, T cells expanded in the presence of siRNA exhibited high cytotoxic activities against autologous tumours as compared to control TIL. Notably, clinical trials have shown promising results with TIL therapy in patients with melanoma [111]. Empowering T cell function by blocking PD-1 expression could easily be adopted into ACT protocols. Moreover, potential resistance to PD-1 therapy may be prevented by silencing additional T cell inhibitory molecules such as TIM-3, LAG-3, and TIGIT. In line with this notion, dual inhibition of IDO and PD-L1 or PD-1 in T cells resulted in more enhanced T cell responses in vitro when compared to mono-gene silencing [121].

In addition to TIL, T cells engineered to express defined TCRs specific for tumour antigens or chimeric antigen receptors (CAR) hold great promise for the treatment of metastatic cancers [112]. CD19-targeting CAR T cells are the first gene therapy drug approved by the U.S. Food and Drug Administration for the treatment of B-cell lymphomas [122]. Although CAR-based ACTs were clinically successful in the treatment of B cell malignancies as documented in multiple independent clinical trials, so far the responses in solid tumours are still limited. The immunosuppressive environment in solid tumours, in conjunction with the expression of inhibitory receptors on the engineered T cells, are thought to negatively influence the efficacy of CAR-T cells. Additionally, in vitro-expanded T cells upregulate the expression of PD-1 that could be suppressed by siRNA [84]. A recent study demonstrated that CAR-T cells transfected with siRNAs targeting PD-1 alone or in combination with siRNA targeting CTLA-4 produced more effector cytokines and showed a significant increase in cytotoxicity towards melanoma cells expressing PD-L1 as compared to control cells [123].

As mentioned above, certain proteins involved in the regulation TCR signalling are surely good targets for inhibition by RNAi technology. For example, the E3 ubiquitin ligase Cbl-b and the src homology 2 domain containing protein tyrosine phosphatase (SHP-1) negatively regulate TCR activation [124]. Both proteins are cytosolic, and therefore not amenable to antibody-mediated therapies. Like CTLA-4 knockout mice, mice deficient in *cblb* gene are very susceptible to spontaneous and antigen-induced experimental autoimmune diseases [125]. Therefore, inhibition of Cbl-b expression in T cells may enhance their antitumor potency. In this respect, silencing of Cbl-b expression in primary murine CD8+ T cells with siRNAs via nucleofection, followed by adoptive transfer of the cells into recipient mice enhanced the effects of an anti-cancer vaccine [126]. SHP-1 is a negative regulator of antigen dependent activation and proliferation of T cells in part by reducing T cell/DC interactions [127]. RNAi inhibition of SHP-1 in T cells enhanced the anti-tumour activity of adoptively transferred T cells [128]. These two examples show that targeting cytosolic inhibitors involved in TCR tuning can enhance or restored T cell function. Although we do not yet know which combination therapies are optimal for clinical translation, the obtained pre-clinical data with siRNAs are very encouraging and should overcome some of the obstacles associated with adoptive immunotherapy for cancer patients.

In addition to ex vivo delivery, several studies have shown that siRNAs can be delivered to immune cells in vivo. For example, antibody fragment fused to protamine or to other nucleic acid-binding peptides have been used to deliver siRNAs and knock down gene expression in tumours, disseminated blood cells and organs such as the lung [17]. Moreover, siRNAs were targeted to 4-1BB-expressiong CD8+ T cells by conjugation to a 4-1BB-binding oligonucleotide aptamer [129]. 4-1BB is a major immune stimulatory receptor transiently expressed on activated CD8+ T cells [130]. The data indicate that 4-1BB aptamer delivery of CD25 siRNA to in vivo activated CD8+ T cells is efficient and specific, leading to enhanced memory differentiation and improved control of tumour growth. Overall, these studies show that receptor-directed antibodies or aptamers can deliver siRNAs to circulating CD4+ and CD8+ T cells. Additionally, the newly engineered nanoparticle formulations like those developed by Peer’s group should facilitate the systemic delivery of siRNAs to T lymphocytes [131].

## 11. Conclusions

The major goal of vaccination against cancer is the generation of high avidity antigen-specific CTLs and long-lived T memory cells. While the current DC cancer vaccines induced antigen-specific immune responses, the duration and magnitude of these responses are weak and clinical benefits have been limited. The knockdown of inhibitory signals by siRNAs is a viable method to make DCs- or T cell-mediated immunotherapies more effective. Most of the targeted inhibitory molecules have been successful in that their inhibition by siRNAs resulted in strong CTL responses. What was remarkable was the objective clinical responses obtained with either IDO- or SOCS-1-silenced DC vaccine. Because of the manufacturing simplicity, the introduction of chemically made siRNAs or shRNAs into DCs along with mRNA encoding TAAs or neoantigens through electroporation is a straightforward strategy that is not associated with additional manufacturing costs. Moreover, a variety of lipid-based siRNA delivery methods and chemical modifications offer an alternative strategy to electroporation, in particular self-delivered siRNAs. These chemically modified molecules can enter immune cells without needing a delivery reagent and can be produced under GMP conditions. The effective targeting of siRNAs to circulating blood T cells suggests that siRNAs could be tailored to provide clinical benefits as cancer immunotherapies. Additionally, nanocarriers are valuable for potentiating the simultaneous delivery of antigens, adjuvants and siRNAs to DCs in situ. Overall, the discussed data demonstrate that it is feasible to use siRNAs to boost DC immunogenic function and T cell cytotoxic function and therefore they should lay the groundwork for designing novel immunotherapeutic strategies for the treatment of advanced cancers.
